# (*Z*)-1,2-Dichloro-1,2-bis­(3-chloro­quinoxalin-2-yl)ethene

**DOI:** 10.1107/S1600536810054322

**Published:** 2011-01-08

**Authors:** Hoong-Kun Fun, Jia Hao Goh, Annada C. Maity, Shyamaprosad Goswami

**Affiliations:** a X-ray Crystallography Unit, School of Physics, Universiti Sains Malaysia, 11800 USM, Penang, Malaysia; b Department of Chemistry, Bengal Engineering and Science University, Shibpur, Howrah 711 103, India

## Abstract

The title compound, C_18_H_8_Cl_4_N_4_, exists in a *cis* configuration with respect to the bridging C=C bond. The two essentially planar quinoxaline ring systems [maximum deviations = 0.012 (1) and 0.022 (1) Å] are inclined at an angle of 59.84 (3). In the crystal, adjacent mol­ecules are linked into chains propagating along [001] *via* inter­molecular C—H⋯N hydrogen bonds. Weak inter­molecular π–π [centroid–centroid distance = 3.6029 (7)°] and C—H⋯π inter­actions are also observed.

## Related literature

For general background to and applications of the title compound, see: Fun *et al.* (2009 ▶); Goswami *et al.* (2007 ▶). For closely related structures, see: Fun *et al.* (2009 ▶); Goswami *et al.* (2007 ▶). For the stability of the temperature controller used in the data collection, see: Cosier & Glazer (1986 ▶).
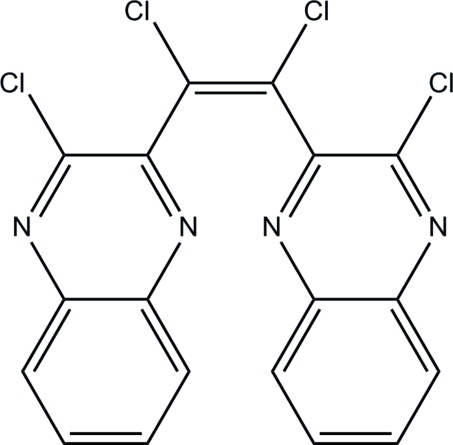


## Experimental

### 

#### Crystal data

C_18_H_8_Cl_4_N_4_
*M* *_r_* = 422.08
Monoclinic, 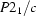

*a* = 19.0972 (5) Å
*b* = 10.9883 (3) Å
*c* = 8.1905 (2) Å
β = 90.782 (1)°
*V* = 1718.58 (8) Å^3^
*Z* = 4
Mo *K*α radiation
μ = 0.70 mm^−1^
*T* = 100 K
0.79 × 0.22 × 0.10 mm

#### Data collection

Bruker SMART APEXII CCD area-detector diffractometer
Absorption correction: multi-scan (*SADABS*; Bruker, 2009 ▶) *T*_min_ = 0.608, *T*_max_ = 0.933
72483 measured reflections
8778 independent reflections
6556 reflections with *I* > 2σ(*I*)
*R*_int_ = 0.061

#### Refinement

*R*[*F* ^2^ > 2σ(*F* ^2^)] = 0.042
*wR*(*F* ^2^) = 0.119
*S* = 1.11
8778 reflections
267 parameters
All H-atom parameters refined
Δρ_max_ = 0.59 e Å^−3^
Δρ_min_ = −0.35 e Å^−3^

### 

Data collection: *APEX2* (Bruker, 2009 ▶); cell refinement: *SAINT* (Bruker, 2009 ▶); data reduction: *SAINT*; program(s) used to solve structure: *SHELXTL* (Sheldrick, 2008 ▶); program(s) used to refine structure: *SHELXTL*; molecular graphics: *SHELXTL*; software used to prepare material for publication: *SHELXTL* and *PLATON* (Spek, 2009 ▶).

## Supplementary Material

Crystal structure: contains datablocks global, I. DOI: 10.1107/S1600536810054322/sj5082sup1.cif

Structure factors: contains datablocks I. DOI: 10.1107/S1600536810054322/sj5082Isup2.hkl

Additional supplementary materials:  crystallographic information; 3D view; checkCIF report

## Figures and Tables

**Table 1:** Hydrogen-bond geometry (Å, °) *Cg*1 is the centroid of the C13–C18 ring.

*D*—H⋯*A*	*D*—H	H⋯*A*	*D*⋯*A*	*D*—H⋯*A*
C17—H17⋯N2^i^	0.995 (17)	2.454 (17)	3.2609 (16)	137.8 (13)
C16—H16⋯*Cg*1^ii^	0.97 (2)	3.00 (2)	3.9664 (16)	176.0 (16)

Symmetry codes: (i) 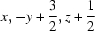; (ii) 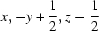.

**Fig. 1.**
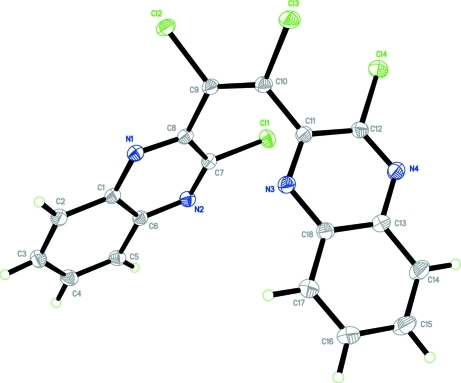
The molecular structure of the title compound, showing 50% probability displacement ellipsoids for non-H atoms and the atom-numbering scheme.

**Fig. 2.**
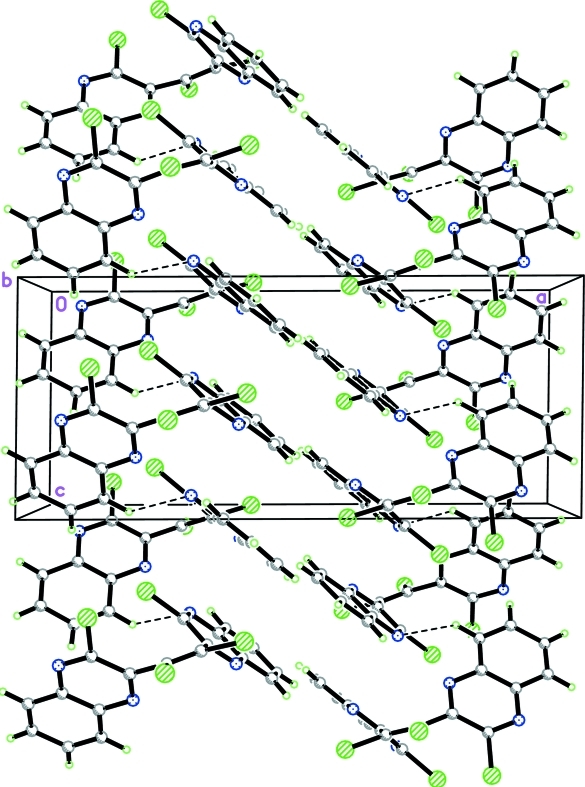
The crystal structure of the title compound, viewed along the b axis, showing a pair of 1D chains propagating along the c axis. Intermolecular hydrogen bonds are shown as dashed lines.

## supplementary crystallographic information

### Comment

Halogen substituted heterocyclic compounds are of great importance due to their broad spectrum of use in organic chemistry. Recently a series of trichloromethyl substituted heterocyclic compounds have been synthesized by us in good yield using *N*-chlorosuccinimide (NCS) and triphenylphosphine (PPh_3_) in carbon tetrachloride (Fun *et al.*, 2009; Goswami *et al.*, 2007). Here we report the results of X-ray crystallographic studies of the supramolecular self-assembly of a chlorine-substituted heterocyclic compound to show its possible choice of polymer formation by self-assembly. Reaction of 2-chloro-3-trichloromethylquinoxaline with Co(I)(PPh_3_)_3_Cl results in the formation of the title compound.

The title compound (Fig. 1) exists in a *cis* configuration with respect to the bridging C9═C10 bond [bond length of C9═C10 = 1.3374 (16) Å and torsion angle of C8–C9–C10–C11 = -0.3 (2)°]. The two quinoxaline ring systems [(C1–C8/N1/N2) & (C11–C18/N3/N4)] are essentially planar, with maximum deviations of 0.022 (1) Å at atom C8 and -0.012 (1) Å at atom C11, respectively. An interplanar angle of 59.84 (3)° is formed between the two quinoxaline ring systems, indicating the molecule is not planar. All geometric parameters are consistent to those observed in closely related structures (Goswami *et al.*, 2007; Fun *et al.*, 2009).

In the crystal packing, intermolecular C17—H17···N2 hydrogen bonds (Table 1) link adjacent molecules into one-dimensional chains in an anti-parallel manner along the *c* axis (Fig. 2). Further stabilization of the crystal packing is provided by weak intermolecular C16—H16···*Cg*1 interactions (Table 1) as well as intermolecular *Cg*2···*Cg*3 interactions [3.6029 (7) Å; symmetry code: x, -y+3/2, z-1/2] where *Cg*1, *Cg*2 are the centroids of the C13–C18 and C1–C6 benzene rings, respectively, and *Cg*3 is the centroid of C1/C6–C8/N1/N2 pyrazine ring.

### Experimental

2-Chloro-3-trichloromethylquinoxaline (1 mmol) was dissolved in dry benzene (30 ml). The anhydrous green coloured Co(I)(PPh_3_)_3_Cl (2.5 mmol) catalyst was added to the reaction mixture with stirring at room temperature under nitrogen atmosphere. After 30 minutes, the colour of the reaction mixture changed from green to blue. The reaction mixture was then heated under reflux condition for 2-3 h. The solvent was evaporated to dryness. The residue was then worked up with water and the organic part was extracted with chloroform. The organic layer was dried (Na_2_SO_4_) and concentrated. Column chromatography of the crude product on silica gel and elution with methanol in chloroform afforded the title compound. Single crystals were grown by slow evaporation of a 1:1 solution of CHCl_3_ and methanol.

### Refinement

All H atoms were located from a difference Fourier map, and allowed to refine freely with range of C—H = 0.89 (2)–0.994 (18) Å. The reflection (100) was omitted as the intensity was affected by the beam backstop.

### Crystal data

**Table:**

C_18_H_8_Cl_4_N_4_	*F*(000) = 848
*M**_r_* = 422.08	*D*_x_ = 1.631 Mg m^−^^3^
Monoclinic, *P*2_1_/*c*	Mo *K*α radiation, λ = 0.71073 Å
Hall symbol: -P 2ybc	Cell parameters from 9887 reflections
*a* = 19.0972 (5) Å	θ = 2.1–37.2°
*b* = 10.9883 (3) Å	µ = 0.70 mm^−^^1^
*c* = 8.1905 (2) Å	*T* = 100 K
β = 90.782 (1)°	Block, yellow
*V* = 1718.58 (8) Å^3^	0.79 × 0.22 × 0.10 mm
*Z* = 4	

### Data collection

**Table:**

Bruker SMART APEXII CCD area-detector diffractometer	8778 independent reflections
Radiation source: fine-focus sealed tube	6556 reflections with *I* > 2σ(*I*)
graphite	*R*_int_ = 0.061
φ and ω scans	θ_max_ = 37.2°, θ_min_ = 2.1°
Absorption correction: multi-scan (*SADABS*; Bruker, 2009)	*h* = −32→32
*T*_min_ = 0.608, *T*_max_ = 0.933	*k* = −18→18
72483 measured reflections	*l* = −13→13

### Refinement

**Table:**

Refinement on *F*^2^	Primary atom site location: structure-invariant direct methods
Least-squares matrix: full	Secondary atom site location: difference Fourier map
*R*[*F*^2^ > 2σ(*F*^2^)] = 0.042	Hydrogen site location: inferred from neighbouring sites
*wR*(*F*^2^) = 0.119	All H-atom parameters refined
*S* = 1.11	*w* = 1/[σ^2^(*F*_o_^2^) + (0.060*P*)^2^ + 0.1538*P*] where *P* = (*F*_o_^2^ + 2*F*_c_^2^)/3
8778 reflections	(Δ/σ)_max_ = 0.001
267 parameters	Δρ_max_ = 0.59 e Å^−^^3^
0 restraints	Δρ_min_ = −0.35 e Å^−^^3^

### Special details

**Table:**

Experimental. The crystal was placed in the cold stream of an Oxford Cryosystems Cobra open-flow nitrogen cryostat (Cosier & Glazer, 1986) operating at 100.0 (1)K.
Geometry. All esds (except the esd in the dihedral angle between two l.s. planes) are estimated using the full covariance matrix. The cell esds are taken into account individually in the estimation of esds in distances, angles and torsion angles; correlations between esds in cell parameters are only used when they are defined by crystal symmetry. An approximate (isotropic) treatment of cell esds is used for estimating esds involving l.s. planes.
Refinement. Refinement of F^2^ against ALL reflections. The weighted R-factor wR and goodness of fit S are based on F^2^, conventional R-factors R are based on F, with F set to zero for negative F^2^. The threshold expression of F^2^ > 2sigma(F^2^) is used only for calculating R-factors(gt) etc. and is not relevant to the choice of reflections for refinement. R-factors based on F^2^ are statistically about twice as large as those based on F, and R- factors based on ALL data will be even larger.

### Fractional atomic coordinates and isotropic or equivalent isotropic displacement parameters (Å^2^)

**Table:**

	*x*	*y*	*z*	*U*_iso_*/*U*_eq_	
Cl1	0.230183 (15)	0.56482 (3)	−0.19687 (4)	0.01973 (7)	
Cl2	0.408666 (15)	0.33929 (3)	−0.01835 (4)	0.02087 (7)	
Cl3	0.277057 (17)	0.19701 (3)	0.08651 (5)	0.02505 (8)	
Cl4	0.139656 (17)	0.28579 (3)	−0.12100 (4)	0.02431 (7)	
N1	0.39159 (5)	0.59732 (9)	0.11036 (12)	0.01616 (17)	
N2	0.30364 (5)	0.74383 (9)	−0.08414 (12)	0.01681 (17)	
N3	0.21167 (5)	0.49059 (9)	0.24250 (12)	0.01686 (17)	
N4	0.08344 (5)	0.42781 (9)	0.09017 (13)	0.01902 (19)	
C1	0.40036 (6)	0.72039 (10)	0.10598 (14)	0.01538 (19)	
C2	0.45443 (6)	0.77568 (11)	0.20007 (15)	0.0180 (2)	
C3	0.46254 (7)	0.89969 (11)	0.19571 (16)	0.0202 (2)	
C4	0.41789 (7)	0.97290 (11)	0.09745 (16)	0.0205 (2)	
C5	0.36555 (7)	0.92203 (10)	0.00467 (15)	0.0181 (2)	
C6	0.35580 (6)	0.79460 (10)	0.00883 (14)	0.01560 (19)	
C7	0.29618 (6)	0.62669 (10)	−0.07490 (14)	0.01589 (19)	
C8	0.33959 (6)	0.55012 (10)	0.02467 (14)	0.01502 (18)	
C9	0.33274 (6)	0.41522 (10)	0.03069 (15)	0.01644 (19)	
C10	0.27542 (6)	0.35370 (10)	0.07344 (15)	0.0173 (2)	
C11	0.20886 (6)	0.41146 (10)	0.12282 (14)	0.01590 (19)	
C12	0.14274 (6)	0.38222 (10)	0.04606 (14)	0.0173 (2)	
C13	0.08526 (6)	0.50918 (11)	0.21677 (15)	0.0181 (2)	
C14	0.02225 (7)	0.56163 (13)	0.26997 (17)	0.0240 (2)	
C15	0.02406 (8)	0.64421 (14)	0.39522 (19)	0.0281 (3)	
C16	0.08807 (8)	0.67632 (13)	0.47183 (19)	0.0275 (3)	
C17	0.15010 (7)	0.62649 (12)	0.42128 (16)	0.0230 (2)	
C18	0.14972 (6)	0.54162 (10)	0.29197 (14)	0.01708 (19)	
H2	0.4818 (9)	0.7241 (16)	0.265 (2)	0.026 (4)*	
H3	0.4954 (10)	0.9394 (17)	0.253 (2)	0.039 (5)*	
H4	0.4232 (9)	1.0550 (17)	0.094 (2)	0.028 (5)*	
H5	0.3373 (9)	0.9670 (16)	−0.066 (2)	0.027 (4)*	
H14	−0.0216 (9)	0.5407 (16)	0.215 (2)	0.030 (5)*	
H15	−0.0164 (12)	0.6781 (18)	0.435 (3)	0.041 (6)*	
H16	0.0870 (10)	0.7361 (18)	0.559 (3)	0.035 (5)*	
H17	0.1949 (9)	0.6476 (16)	0.478 (2)	0.026 (4)*	

### Atomic displacement parameters (Å^2^)

**Table:**

	*U*^11^	*U*^22^	*U*^33^	*U*^12^	*U*^13^	*U*^23^
Cl1	0.01985 (13)	0.01898 (12)	0.02025 (13)	−0.00230 (9)	−0.00389 (10)	0.00064 (9)
Cl2	0.01669 (12)	0.01806 (12)	0.02796 (15)	0.00393 (9)	0.00359 (10)	0.00006 (10)
Cl3	0.02286 (14)	0.01315 (12)	0.03931 (18)	0.00158 (9)	0.00726 (12)	0.00199 (11)
Cl4	0.02400 (14)	0.02459 (14)	0.02439 (15)	−0.00171 (11)	0.00284 (11)	−0.00901 (11)
N1	0.0155 (4)	0.0167 (4)	0.0163 (4)	0.0010 (3)	0.0017 (3)	0.0000 (3)
N2	0.0176 (4)	0.0160 (4)	0.0169 (4)	−0.0001 (3)	0.0009 (3)	0.0005 (3)
N3	0.0165 (4)	0.0152 (4)	0.0190 (4)	0.0001 (3)	0.0029 (3)	0.0008 (3)
N4	0.0170 (4)	0.0209 (4)	0.0192 (5)	0.0002 (3)	0.0011 (4)	−0.0016 (4)
C1	0.0148 (4)	0.0158 (4)	0.0155 (5)	0.0010 (3)	0.0024 (4)	0.0005 (3)
C2	0.0162 (5)	0.0204 (5)	0.0174 (5)	0.0008 (4)	−0.0001 (4)	−0.0016 (4)
C3	0.0189 (5)	0.0204 (5)	0.0213 (5)	−0.0033 (4)	0.0009 (4)	−0.0026 (4)
C4	0.0230 (6)	0.0158 (5)	0.0227 (6)	−0.0016 (4)	0.0030 (4)	−0.0008 (4)
C5	0.0205 (5)	0.0151 (4)	0.0186 (5)	0.0011 (4)	0.0015 (4)	0.0007 (4)
C6	0.0145 (4)	0.0163 (4)	0.0161 (5)	0.0006 (3)	0.0022 (4)	−0.0002 (3)
C7	0.0149 (4)	0.0167 (4)	0.0161 (5)	0.0007 (3)	0.0005 (4)	0.0007 (4)
C8	0.0145 (4)	0.0149 (4)	0.0157 (5)	0.0013 (3)	0.0021 (4)	0.0007 (3)
C9	0.0158 (5)	0.0145 (4)	0.0190 (5)	0.0017 (3)	0.0018 (4)	0.0005 (4)
C10	0.0171 (5)	0.0139 (4)	0.0210 (5)	0.0012 (3)	0.0027 (4)	0.0008 (4)
C11	0.0158 (5)	0.0137 (4)	0.0183 (5)	0.0005 (3)	0.0028 (4)	0.0016 (3)
C12	0.0183 (5)	0.0164 (5)	0.0174 (5)	−0.0004 (4)	0.0019 (4)	−0.0017 (4)
C13	0.0181 (5)	0.0189 (5)	0.0172 (5)	0.0021 (4)	0.0012 (4)	0.0001 (4)
C14	0.0178 (5)	0.0288 (6)	0.0255 (6)	0.0051 (4)	0.0014 (5)	−0.0020 (5)
C15	0.0243 (6)	0.0317 (7)	0.0286 (7)	0.0078 (5)	0.0054 (5)	−0.0047 (5)
C16	0.0282 (7)	0.0267 (6)	0.0280 (7)	0.0018 (5)	0.0068 (5)	−0.0085 (5)
C17	0.0221 (6)	0.0228 (5)	0.0241 (6)	−0.0016 (4)	0.0024 (5)	−0.0059 (5)
C18	0.0177 (5)	0.0157 (4)	0.0179 (5)	0.0007 (4)	0.0021 (4)	−0.0003 (4)

### Geometric parameters (Å, °)

**Table:**

Cl1—C7	1.7362 (11)	C4—H4	0.908 (18)
Cl2—C9	1.7250 (12)	C5—C6	1.4130 (15)
Cl3—C10	1.7253 (11)	C5—H5	0.927 (18)
Cl4—C12	1.7310 (12)	C7—C8	1.4289 (15)
N1—C8	1.3149 (15)	C8—C9	1.4889 (15)
N1—C1	1.3632 (14)	C9—C10	1.3374 (16)
N2—C7	1.2974 (15)	C10—C11	1.4819 (16)
N2—C6	1.3646 (15)	C11—C12	1.4393 (16)
N3—C11	1.3108 (15)	C13—C14	1.4086 (17)
N3—C18	1.3754 (15)	C13—C18	1.4146 (17)
N4—C12	1.2941 (16)	C14—C15	1.370 (2)
N4—C13	1.3692 (16)	C14—H14	0.974 (18)
C1—C6	1.4156 (15)	C15—C16	1.411 (2)
C1—C2	1.4169 (16)	C15—H15	0.92 (2)
C2—C3	1.3720 (17)	C16—C17	1.3739 (19)
C2—H2	0.934 (18)	C16—H16	0.97 (2)
C3—C4	1.4153 (18)	C17—C18	1.4111 (17)
C3—H3	0.89 (2)	C17—H17	0.994 (18)
C4—C5	1.3669 (17)		
			
C8—N1—C1	117.99 (10)	C10—C9—Cl2	120.67 (9)
C7—N2—C6	116.94 (10)	C8—C9—Cl2	113.54 (8)
C11—N3—C18	117.65 (10)	C9—C10—C11	124.28 (10)
C12—N4—C13	116.81 (10)	C9—C10—Cl3	120.43 (9)
N1—C1—C6	120.87 (10)	C11—C10—Cl3	115.17 (8)
N1—C1—C2	119.99 (10)	N3—C11—C12	120.10 (10)
C6—C1—C2	119.13 (10)	N3—C11—C10	117.49 (10)
C3—C2—C1	119.56 (11)	C12—C11—C10	122.40 (10)
C3—C2—H2	123.7 (11)	N4—C12—C11	123.88 (11)
C1—C2—H2	116.7 (11)	N4—C12—Cl4	115.93 (9)
C2—C3—C4	120.80 (11)	C11—C12—Cl4	120.14 (9)
C2—C3—H3	123.4 (12)	N4—C13—C14	119.24 (11)
C4—C3—H3	115.8 (12)	N4—C13—C18	120.48 (11)
C5—C4—C3	120.93 (11)	C14—C13—C18	120.27 (11)
C5—C4—H4	118.1 (11)	C15—C14—C13	119.33 (12)
C3—C4—H4	121.0 (11)	C15—C14—H14	121.1 (11)
C4—C5—C6	119.17 (11)	C13—C14—H14	119.5 (11)
C4—C5—H5	122.9 (11)	C14—C15—C16	120.76 (13)
C6—C5—H5	117.9 (11)	C14—C15—H15	121.1 (13)
N2—C6—C5	119.15 (10)	C16—C15—H15	118.0 (13)
N2—C6—C1	120.45 (10)	C17—C16—C15	120.76 (13)
C5—C6—C1	120.40 (10)	C17—C16—H16	121.3 (11)
N2—C7—C8	123.65 (10)	C15—C16—H16	117.9 (11)
N2—C7—Cl1	115.75 (8)	C16—C17—C18	119.60 (12)
C8—C7—Cl1	120.58 (8)	C16—C17—H17	120.4 (10)
N1—C8—C7	120.03 (10)	C18—C17—H17	120.0 (10)
N1—C8—C9	116.17 (10)	N3—C18—C17	119.66 (11)
C7—C8—C9	123.69 (10)	N3—C18—C13	121.06 (10)
C10—C9—C8	125.76 (10)	C17—C18—C13	119.27 (11)
			
C8—N1—C1—C6	−1.53 (16)	C8—C9—C10—Cl3	−176.06 (9)
C8—N1—C1—C2	178.18 (11)	Cl2—C9—C10—Cl3	2.01 (15)
N1—C1—C2—C3	−179.56 (11)	C18—N3—C11—C12	1.06 (16)
C6—C1—C2—C3	0.15 (17)	C18—N3—C11—C10	−178.07 (10)
C1—C2—C3—C4	−0.48 (19)	C9—C10—C11—N3	−54.58 (17)
C2—C3—C4—C5	0.10 (19)	Cl3—C10—C11—N3	121.43 (10)
C3—C4—C5—C6	0.62 (19)	C9—C10—C11—C12	126.31 (13)
C7—N2—C6—C5	−179.23 (11)	Cl3—C10—C11—C12	−57.69 (14)
C7—N2—C6—C1	1.88 (16)	C13—N4—C12—C11	0.56 (17)
C4—C5—C6—N2	−179.84 (11)	C13—N4—C12—Cl4	−176.74 (9)
C4—C5—C6—C1	−0.94 (18)	N3—C11—C12—N4	−1.63 (18)
N1—C1—C6—N2	−0.85 (17)	C10—C11—C12—N4	177.46 (11)
C2—C1—C6—N2	179.44 (11)	N3—C11—C12—Cl4	175.56 (9)
N1—C1—C6—C5	−179.73 (11)	C10—C11—C12—Cl4	−5.35 (16)
C2—C1—C6—C5	0.56 (17)	C12—N4—C13—C14	−179.97 (12)
C6—N2—C7—C8	−0.66 (17)	C12—N4—C13—C18	0.90 (17)
C6—N2—C7—Cl1	−179.12 (9)	N4—C13—C14—C15	−179.18 (13)
C1—N1—C8—C7	2.74 (16)	C18—C13—C14—C15	−0.05 (19)
C1—N1—C8—C9	178.96 (10)	C13—C14—C15—C16	−0.5 (2)
N2—C7—C8—N1	−1.74 (18)	C14—C15—C16—C17	0.7 (2)
Cl1—C7—C8—N1	176.65 (9)	C15—C16—C17—C18	−0.4 (2)
N2—C7—C8—C9	−177.66 (11)	C11—N3—C18—C17	179.47 (11)
Cl1—C7—C8—C9	0.73 (16)	C11—N3—C18—C13	0.36 (16)
N1—C8—C9—C10	124.93 (13)	C16—C17—C18—N3	−179.24 (12)
C7—C8—C9—C10	−59.01 (18)	C16—C17—C18—C13	−0.11 (19)
N1—C8—C9—Cl2	−53.26 (13)	N4—C13—C18—N3	−1.41 (18)
C7—C8—C9—Cl2	122.81 (11)	C14—C13—C18—N3	179.47 (11)
C8—C9—C10—C11	−0.3 (2)	N4—C13—C18—C17	179.47 (11)
Cl2—C9—C10—C11	177.81 (9)	C14—C13—C18—C17	0.35 (18)

### Hydrogen-bond geometry (Å, °)

**Table:**

Cg1 is the centroid of the C13–C18 ring.

**Table:**

*D*—H···*A*	*D*—H	H···*A*	*D*···*A*	*D*—H···*A*
C17—H17···N2^i^	0.995 (17)	2.454 (17)	3.2609 (16)	137.8 (13)
C16—H16···Cg1^ii^	0.97 (2)	3.00 (2)	3.9664 (16)	176.0 (16)

Symmetry codes: (i) *x*, −*y*+3/2, *z*+1/2; (ii) *x*, −*y*+1/2, *z*−1/2.
